# Real-world histopathological approach to malignancy of undefined primary origin (MUO) to diagnose cancers of unknown primary (CUPs)

**DOI:** 10.1007/s00428-022-03435-z

**Published:** 2022-11-08

**Authors:** Alberto Pisacane, Eliano Cascardi, Enrico Berrino, Alessio Polidori, Ivana Sarotto, Laura Casorzo, Mara Panero, Carla Boccaccio, Federica Verginelli, Silvia Benvenuti, Miriam Dellino, Paolo Comoglio, Filippo Montemurro, Elena Geuna, Caterina Marchiò, Anna Sapino

**Affiliations:** 1 Candiolo Cancer Institute, FPO-IRCCS, 10060 Candiolo, Turin, Italy; 2 Department of Medical Sciences, University of Turin, 10100 Turin, Italy; 3 Department of Oncology, University of Turin Medical School, 10060 Candiolo, Turin, Italy; 4 Department of Biomedical Sciences and Human Oncology, University of Bari “Aldo Moro”, Bari, Italy; 5 IFOM, FIRC Institute of Molecular Oncology, 20019 Milan, Italy

**Keywords:** Malignancies of undefined primary origin (MUO), Cancer of unknown primary (CUP), Immunohistochemistry, Tissue of origin, Carcinoma

## Abstract

The aim of this study is to envisage a streamlined pathological workup to rule out CUPs in patients presenting with MUOs. Sixty-four MUOs were classified using standard histopathology. Clinical data, immunocytochemical markers, and results of molecular analysis were recorded. MUOs were histologically subdivided in clear-cut carcinomas (40 adenocarcinomas, 11 squamous, and 3 neuroendocrine carcinomas) and unclear-carcinoma features (5 undifferentiated and 5 sarcomatoid tumors). Cytohistology of 7/40 adenocarcinomas suggested an early metastatic cancer per se. In 33/40 adenocarcinomas, CK7/CK20 expression pattern, gender, and metastasis sites influenced tissue-specific marker selection. In 23/40 adenocarcinomas, a “putative-immunophenotype” of tissue of origin addressed clinical-diagnostic examinations, identifying 9 early metastatic cancers. Cell lineage markers were used to confirm squamous and neuroendocrine differentiation. Pan-cytokeratins were used to confirm the epithelial nature of poorly differentiated tumors, followed by tissue and cell lineage markers, which identified one melanoma. In total, 47/64 MUOs (73.4%) were confirmed CUP. Molecular analysis, feasible in 37/47 CUPs (78.7%), had no diagnostic impact. Twenty CUP patients, mainly with squamous carcinomas and adenocarcinomas with putative-gynecologic-immunophenotypes, presented with only lymph node metastases and had longer median time to progression and overall survival (< 0.001), compared with patients with other metastatic patterns. We propose a simplified histology-driven workup which could efficiently rule out CUPs and identify early metastatic cancer.

**Supplementary Information:**

The online version contains supplementary material available at 10.1007/s00428-022-03435-z.

## Introduction

The finding of a metastatic cancer at the time of first diagnosis is a dramatic event for patients and at the same time a frustrating experience for clinicians. It is currently estimated that, despite many efforts, in about 3% of metastatic patients, the tissue of origin of the neoplastic lesion remains unknown; hence, the patient is regarded as harboring a cancer of unknown primary (CUP) [1]. However, CUP diagnosis requires an extensive clinical, instrumental, and pathological workup before being confirmed [2]. The NICE guidelines distinguish indeed “Malignancy of Undefined primary Origin (MUO)” and “provisional” CUP that is “confirmed” only if “no primary site is detected despite selected initial screen of investigations, specialist review, and further specialized investigations as appropriate” [2]. Regrettably, when receiving cases in consultation or patient-initiated second opinion, the pathologists are not always informed whether the case is a MUO or a provisional CUP. The Royal Collage of Pathologists [3], aligning with NICE recommendations, declared crucial a stepwise approach that uses clinical context, morphology, immunohistochemistry, and, occasionally, other techniques including molecular analysis to confirm or exclude CUP. Gene expression profiling [4] and epigenetic profiling [5] have been introduced de facto with the purpose of unmasking the tissue of origin. Although these studies provided encouraging data, the implementation of such methodologies is far from being prime time in clinical practice, and pathologists are still expected to find the tissue of origin using standard immunohistochemistry (IHC). This pressure leads pathologists to look for the tissue of origin by testing many different tissue-specific markers, which frequently do not resolve the diagnostic problem and waste small specimens, which should otherwise be preserved for high throughput molecular analyses possibly useful for therapeutic decision. The Royal Collage of Pathologists stressed the importance of the histotype to address immunohistochemical analyses correctly. The histotypes considered by ESMO guidelines [6] are well- and moderately-differentiated adenocarcinomas, squamous cell carcinomas, carcinomas with neuroendocrine differentiation, poorly differentiated carcinomas (including poorly differentiated adenocarcinomas), and undifferentiated neoplasms. The same guidelines [6] indicate cytokeratin-7 (CK7) and cytokeratin-20 (CK20) as primary markers in the basic immunohistochemical workup of CUPs, followed by other tissue-specific markers to recognize the tissue of origin. However, recently, Pauli et al. [7] highlighted the challenge in identifying patients with CUP according to ESMO guidelines.

Taking all these issues in mind, we decided to consider the real-world approach followed by pathologists to confirm or to exclude a CUP diagnosis and examine how much standard histology can be of aid in this process. Using the classical cyto-histological criteria of different carcinoma histotypes, we reclassified a retrospective series of MUOs and recorded the IHC tests used to study the tissue of origin. We recognized that adenocarcinomas may show some “flavor” of tissue of origin representative of putative immunophenotypes*.* These latter are suitable to direct targeted investigations to rule out early metastatic cancer. Finally, we analyzed whether CUP histotypes and putative immunophenotypes of adenocarcinomas in the context of distinct clinical presentations could be associated with specific pathways of disease progression and patient survival.

## Materials and methods

### Cohort

Sixty-four patients 38 women (59.4%) and 26 men (40.6%) with an MUO diagnosis were referred to the Candiolo Cancer Institute FPO-IRCCS (Candiolo, Italy) between 2013 and the first trimester of 2021. The samples were obtained using cytological procedures, core biopsies, and excisional biopsy. One patient (AGN43) gave premortem consent to warm autopsy, and multiple samples were obtained [8]. Patients with hematological malignancies were excluded a priori. Symptom-directed endoscopy and additional imaging were performed if required, according to guidelines [9]. Of note, 34/64 patients performed also PET analysis together with other imaging procedures (CT scans). The site of metastases at disease diagnosis was recorded. PET analysis was never resolutive in suggesting the most likely site of origin of the tumor. If only lymph node metastases were present, the number of lymph nodes (single or multiple), the specific lymph node affected, and the lymph node regional localization whether superficial or deep and sub- or supra-diaphragmatics were recorded.

The study was approved by the local ethical committee. Written informed consent was obtained by all patients (AGNOSTOS PROFILING n 010-IRCC-10IIS-15 and upgrade, approved by the ethical committee of FPO-IRCCS Candiolo Cancer Institute). Patients with diagnosis of confirmed CUP were proposed to be enrolled in AGNOSTOS trial (EudraCT Number: 2014–005,018-47), a phase II, randomized, multicenter study to assess the efficacy of nab-paclitaxel-based in association with gemcitabine or carboplatin as first-line therapy.

### Histological and immunohistochemical (IHC) analyses

Slides were reviewed by two pathologists (AP and EC). Similarly to other grading system [9], all cases were graded adopting a scoring system applied to the following parameters: (i) gland formation in any of the architectural variants (e.g., tubular, alveolar, acinar, follicular, papillary) or intracellular mucin in adenocarcinoma, keratinization features in squamous carcinomas, and nests or insular or trabecular growth of cells in NE cancers (score 1: > 75%; score 2: 10–75%; score 3: 1–9%); (ii) mitotic index evaluated in 10 fields of 1 mm^2^ (score 1: 0–5 mitoses; score 2: 6–10 mitoses; score 3: > 11 mitoses); and (iii) nuclear pleomorphism defined as variation in size and shape of nuclei (score 1: minimal nuclear pleomorphism; score 2: moderate nuclear pleomorphism; score 3: marked nuclear pleomorphism). The cut-offs for grading were set as follows: grade 1 score 1–5; grade 2 score 6–7; grade 3 score 8–9.

When cases were received for second opinion with IHC for tissue-specific markers already performed, the slides were reviewed by the local dedicated pathologist (AP), and the IHC was repeated, or new markers were tested if deemed necessary, and the residual material was adequate. Nuclear, cytoplasmatic, or membrane expression of tissue-specific markers and of cell lineage markers were scored, independently from staining intensity, as follows: score 0 =  < 5% of neoplastic cells stained; score 1 = 5–10% of neoplastic cells stained; score 2 = 11–50%; and score 3 > 50% of neoplastic cells stained.

### Molecular analysis

When we initiated the AGNOSTOS trial, we offered to CUP patients the ONCOCARTA Gene Panel version 0.1 (Agena Bioscience, Hamburg, Germany) that includes known druggable hotspot mutations of *ABL1*, *AKT1*, *AKT2*, *BRAF*, *CDK4*, *EGFR*, *ERBB2*, *FGFR1*, *FGFR3*, *FLT3*, *HRAS*, *JAK2*, *KIT*, *KRAS*, *MET*, *NRAS*, *PDGFA*, *PIK3CA*, and *RET* genes.

Cellularity was the parameter considered for specimen adequacy. DNA was extracted GeneRead DNA FFPE Kit (Qiagen, Hilden, Germany) from four 7-µm-thick slides that were hematoxylin and eosin stained and microdissected under a stereomicroscope to enrich for tumor cell content. DNA samples were quantified with spectrophotometric (Nanodrop 1000, Thermo Fisher Scientific, Waltham, MA, USA) and fluorometric (Qubit, Thermo Fisher Scientific, Waltham, MA, USA) assays. Mutational analysis was performed by using the mass spectrometry matrix-assisted laser desorption ionization time-of-flight method with the MassARRAY System (Agena Bioscience, Hamburg, Germany) and the ONCOCARTA as reported above.

### Statistical analysis

Data were analyzed with IBM SPSS Statistics, Version 20.0. (IBM Corp Armonk, NY, USA). Contingency tables with the chi-squared test were applied to infer proportions between groups. Overall survival (OS) was evaluated by the Kaplan–Meier method and analyzed with the log-rank test. Surviving patients were censored at the date of the last follow-up. A *p* < 0.05 was considered statistically significant.

## Results

### MUO histotype definition

Tumor histotype, standard morphological parameters, and grade of the 64 MUOs are reported in Table 1. Fifty-four (84.4%) cases showed clear-cut cytohistological features of carcinoma. Of these, 40 (74%) were classified as adenocarcinoma, 11 squamous (20.3%), and 3 neuroendocrine carcinomas (5.5%). Ten cases (15.6%) did not show clear-cut features of epithelial malignancy; 5 of these (50%) were classified as undifferentiated tumors and 5 as sarcomatoid tumors (50%).

**Table 1:** Histotype, architecture, cell features, and grade of 64 MUO

No of cases (%)	Histotypes	Architectural and cell features	G1 (%)	G2 (%)	G3 (%)
54 (84.4)	Clear-cut features of carcinoma				
40 (74)	Adenocarcinoma	Glandular structures in any of the architectural variants (tubular, alveolar, acinar, follicular, papillary) or intracellular mucin	6 (15)	15 (37.5)	19 (47.5)
11 (20.3)	Squamous cell carcinoma	Solid clusters, sheet-like patterns. Variable keratinization and absence of gland-like structures	0 (0)	4 (36.4)	7 (63.6)
3 (5.5)	Neuroendocrine carcinoma	“Organoid” growth patterns (i.e., trabecular, insular, or sheet-like patterns either singly or in combination). Peripheral palisading may be seen. Round or oval nuclei with “salt and pepper” chromatin	0 (0)	0 (0)	3 (100)
10 (15.6)	No clear-cut features of carcinoma				
5 (50)	Not otherwise specified (NOS)/undifferentiated tumor	Solid growth of epithelioid cells, absence of gland-like structures or keratinization	0 (0)	0 (0)	5 (100)
5 (50)	Sarcomatoid tumors	Solid growth of elongated or pleomorphic cells	0 (0)	0 (0)	5 (100)

Of the 54 cases defined at histology as carcinomas, the majority were either G3 (53.7%) or G2 (37%), while 9.3% were G1 (*p* < 0.01) compared to the undifferentiated and sarcomatoid tumors, which were consistently classified as G3.

### Immunohistochemical workup

#### MUOs with clear-cut carcinoma histology

Adenocarcinomas were the most represented (*n* = 40/64, 62.5%) and required relatively extensive immunohistochemical analysis to formulate the diagnosis of provisional CUP and then exclude the presence of a primary tumor by imaging to reach the diagnosis of confirmed CUP [2, 10]. Women and men were 27 (67.5%) and 13 (32.5%), respectively, with a trend of significance for higher women representation compared with the non-adenocarcinoma CUPs (*p* = 0.087).

**Fig. 1:**
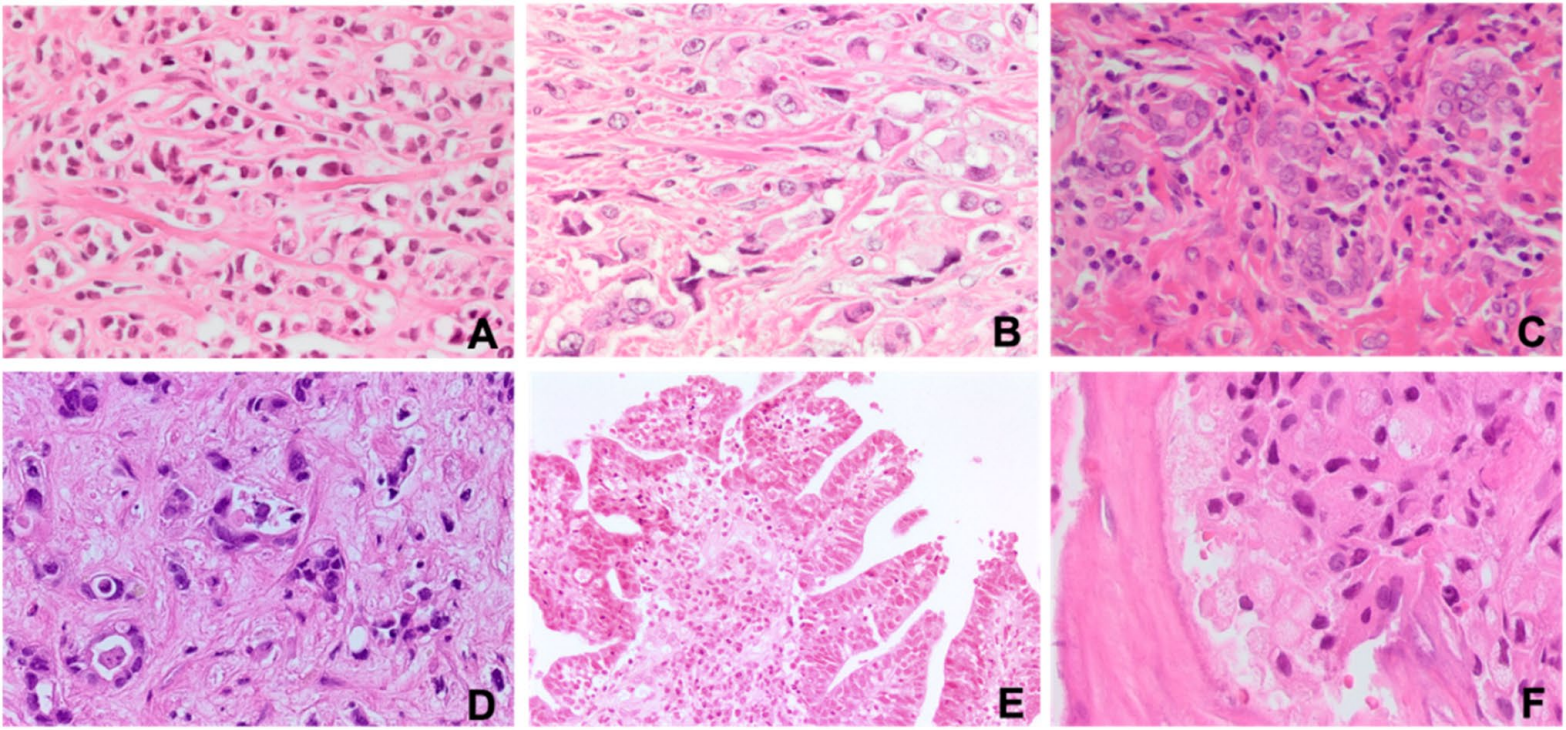
Cytohistological patterns suggestive of tissue of origin. **A** AGN40 liver and **B** EM01/14.15 ovary biopsies of lobular breast cancer metastasis; **C** CUP17/8.15 latero-cervical lymph node biopsy of follicular thyroid carcinoma; **D** AGN336 liver biopsy of intrahepatic cholangiocarcinoma; **E** AGN329 ileo-psoas muscle biopsy of G1 papillary adenocarcinoma metastases; **F** AGN52 bone biopsy of signet ring cell gastric cancer metastasis

Histological revision identified 7/40 (17, 5%) early metastatic adenocarcinomas. Two lobular breast cancers (AGN40 and EM01/14.15, liver (Fig. 1A) and ovary (Fig. 1B) metastases, both resulted CK7 + /CK20 − and estrogen receptor (ER) score 3. Microfollicular structures were identified on deeper histological sections of a single metastasis in a latero-cervical lymph node, diagnosed at another institution as CUP, TTF1 + (CUP17/8.15) (Fig. 1C). Thyroglobulin expression further confirmed the thyroid origin. AGN336 patient was incidentally discovered with lung and liver localizations after a CT scan for SARS-CoV2 infection. The liver core biopsy was sent to our institution for consultation as “adenocarcinoma ER/TTF-1/GATA3/CDX2 negative, possible CUP.” Based on the morphology of well-differentiated glands in sclerotic stroma, a diagnosis of “intrahepatic cholangiocarcinoma” (Fig. 1D) with lung metastases was given, and the patient was treated accordingly. The tumor was CK7 + /CK20 − /ER − and DOG-1 score 1. AGN329 patient presented with a single localization within the ileo-psoas muscle; her biopsy was sent to our service for consultation as CUP, vimentin score 1 + , and calretinin − /SOX10 − /CD10 − . Based on morphology (Fig. 1E), we suspected a gynecologic origin, which was confirmed by ER, PAX8, and WT1 positivity*.* The patient had an endometrial biopsy diagnosed as G1 papillary endometrioid carcinoma of the uterus, and the case was reclassified as early metastatic carcinomas. AGN343 patient had a pseudomyxoma peritonei (CK7 − /CK20 score 3 + /CDX2 score 3/SABT2 score 1, and ER − /PAX8 − /WT1 −); she underwent right and transversal colectomy during debulking, but neither primary cancers nor polyps were identified. Pancreas and stomach were not affected by any lesion. The patient was treated as carrying a tumor of colorectal origin KRAS mutated (see Fig. 6, ONCOCARTA result). In AGN52, biopsy of a bone metastasis showed signet ring cells at histology (Fig. 1F). WT1 and CDX2 showed both score 1 expression, and TTF1 was negative. The histology-driven endoscopy detected a primary gastric adenocarcinoma.

**Fig. 2:**
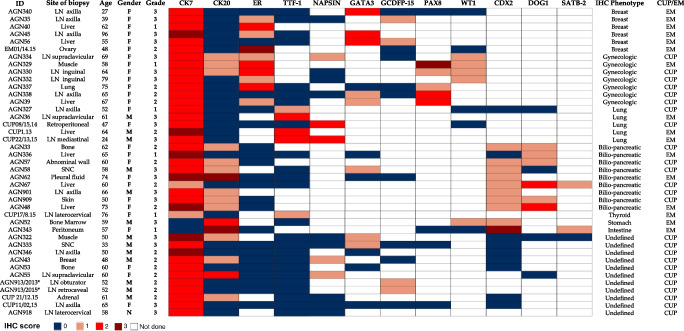
Heat map of tissue-specific marker scores in MUO adenocarcinoma. Heat map depiction of immunohistochemical scores of the most representative tissue-specific markers used in the diagnostic workflow. Data are sorted based on the “putative” immunophenotype (IHC phenotype). Each row represents one case. Columns represent patient identification code (ID), site of biopsy, patient age, patient gender (female: F, male: M), tumor grade (grade 1, grade 2, grade 3), immunohistochemical scores of tissue-specific markers (IHC score): 0 =  < 5% of neoplastic cells stained; 1 = 5–10% of neoplastic cells stained; 2 = 11–50%; 3 =  > 50% of neoplastic cells stained; white fields: not performed; confirmed cancer of unknown primary (CUP)/early metastatic cancers (EM). (AGN913)*two biopsies of the same patients

The remaining 33/40 adenocarcinomas were all tested using anti-CK7/CK20 antibodies. Thirty-eight other IHC markers, used in different combinations by pathologists, were recorded in this set. We selected the immunostaining score values of the 10 most recurrent tissue-specific markers to produce a heatmap (Fig. 2) and to evaluate their impact on ruling out CUP diagnosis. A “putative immunophenotype” of potential tissue of origin was suggested based on the results of tissue-specific markers. Except for CK7, the expression score was generally lower in confirmed CUPs than in non-CUP cases.

In 10/33 cases (30, 3%) (3 women and 7 men), none of the markers was convincing for any “flavor” of tissue of origin (undefined immunophenotype), nor any instrumental examination ratified a primary tumor, directly leading to the definition of confirmed CUPs. One of these patients (AGN913) had biopsies of obturator and retrocaval lymph nodes 2 years apart that maintained the same “undefined profile” (CK7 + /CK20 − /ER − /TTF1 − /GCDFP15 score 1). AGN43 has been described previously [8]. During “warm autopsy,” samples of different anatomical sites of metastases were collected, and 16 different tissue-specific markers were performed, but none of them were useful to define the primary tumor.

In the remaining 23/33 (69.7%) adenocarcinomas, all tested for CK7/CK20, a putative immunophenotype was suggested setting the premises for further diagnostic exploration in related organs. Following imaging and/or endoscopy guided examinations, in 9/33 (27.2%) cases, an early metastatic tumor was found, while in the remaining 14/33 (42.4%), the primary was not found, leading to the diagnosis of confirmed CUP.

All CK7 + /CK20 − and CK7 + /CK20 + biopsies from women were tested for estrogen receptors (ER). CK7 + /CK20 − /ER + immunophenotype per se, independently from the ER IHC score, identified 2 out of the 6 early metastatic breast cancers*.* GATA3 was considered as a breast cancer marker in CK7 + /CK20 − /ER − female adenocarcinomas, and other 2 early metastatic breast cancers were identified. In both CK7 + /CK20 − /ER + (1 case) and CK7 + /CK20 + /ER + (2 cases) adenocarcinomas, PAX8/WT1 expression was performed to rule out gynecological cancers. All cases showing a putative gynecologic immunophenotype were diagnosed as confirmed CUP.

In CK7 + /CK20 − male and in CK7 + /CK20 − /ER − female adenocarcinomas, TTF1 and/or napsin A were the first option, to exclude the lung origin. If TTF1 and or napsin A were positive (4 cases), pathologists generally did not proceed with other markers. Positron emission tomography (PET) and computed tomography (CT) scan confirmed a possible primary lung cancer in 3 patients, and they were studied for treated consequently.

In CK7 + /CK20 + /ER − and in CK7 + /CK20 − cases, CDX2/DOG1 expression suggested a putative biliopancreatic immunophenotype. Two out of seven cases resulted to be early metastatic cholangiocarcinomas.

**Fig. 3:**
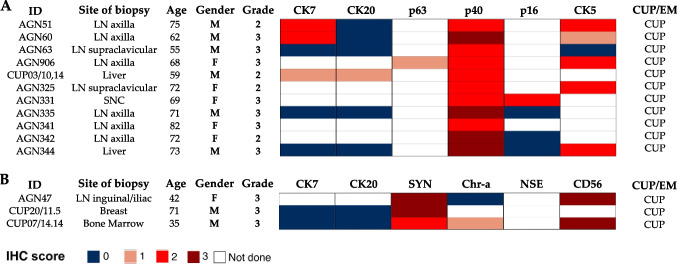
Heat map of tissue-specific marker scores in squamous and neuroendocrine carcinomas. Heat map depiction of immunohistochemical scores of the most representative tissue-specific markers and cell lineage markers used in **A** squamous carcinoma and **B** neuroendocrine carcinoma. Results are not sorted. Each row represents one case. Columns represent patient identification code (ID), site of biopsy, patient age, patient gender (female, male), tumor grade (grade 1, grade 2, grade 3), immunohistochemical scores of tissue-specific markers (IHC score): 0 =  < 5% of neoplastic cells stained; 1 = 5–10% of neoplastic cells stained; 2 = 11–50%; 3 =  > 50% of neoplastic cells stained; white fields: not performed; confirmed cancer of unknown primary (CUP)/early metastatic cancers (EM)

In squamous cell and neuroendocrine carcinomas, 24 and 12 different IHC markers were tested, respectively; none were useful to rule out CUP, but only to confirm the cell lineage differentiation of the tumor (Fig. 3A and B). Only the brain biopsy of AGN331 was p16 positive out of 4 squamous carcinomas tested, suggesting a possible HPV-related cancer of the upper respiratory or genital tract, which was not confirmed at further examinations.

### MUOs with unclear carcinoma histology

**Fig. 4:**
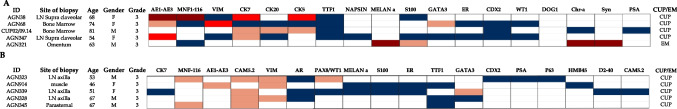
Heat map of tissue-specific marker scores in undifferentiated and sarcomatoid tumors. Heat map depiction of immunohistochemical scores of the most representative tissue-specific markers and cell lineage markers used in **A** undifferentiated and **B** sarcomatoid tumors. Results are not sorted. Each row represents one case. Columns represent patient identification code (ID), site of biopsy, patient age, patient gender (female, male), tumor grade (grade 1, grade 2, grade 3), immunohistochemical scores of tissue-specific markers (IHC score): 0 =  < 5% of neoplastic cells stained; 1 = 5–10% of neoplastic cells stained; 2 = 11–50%; 3 =  > 50% of neoplastic cells stained; white fields: not performed; confirmed cancer of unknown primary (CUP)/early metastatic cancers (EM)

Undifferentiated tumors (2 women and 3 men) were investigated with an extensive panel of cytokeratins to confirm their epithelial nature and then with different tissue-specific and cell lineage markers (18 different markers). Excluding the laparoscopic omental biopsy of an undifferentiated tumor expressing Melan A, S100, chromogranin A, and synaptophysin, which led to the endoscopic identification of an intestinal melanoma with aberrant neuroendocrine differentiation, the other cases lacked any tissue-specific marker and were confirmed as CUPs (Fig. 4A). Sarcomatoid tumors were primarily studied with pan-cytokeratins to exclude not epithelial cancers. Fifteen other markers were used; none of them were useful to identify/suggest a tissue of origin (Fig. 4B).

### Histology-driven algorithm for MUO diagnosis

**Fig. 5:**
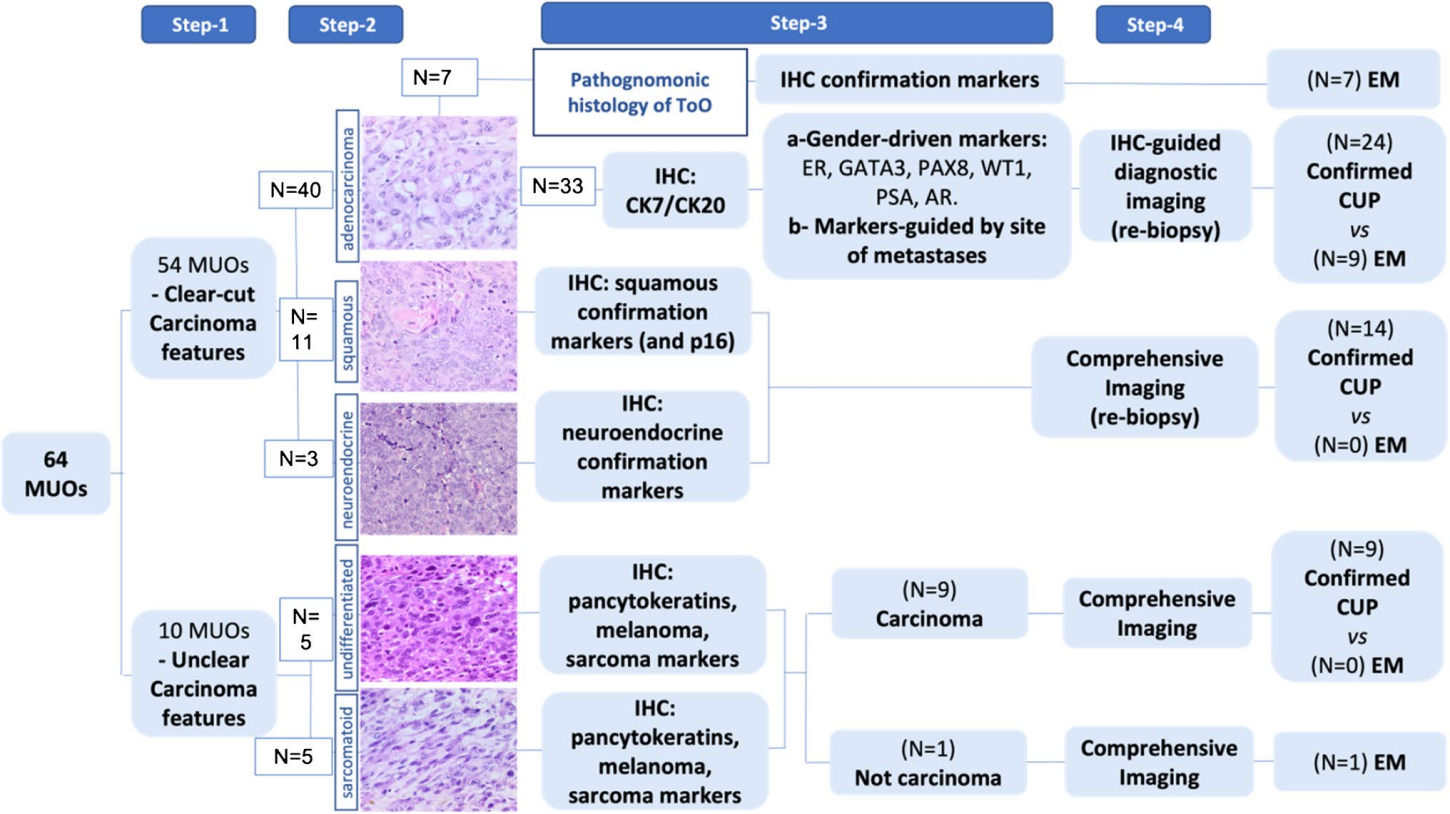
Workup of MUOs. A 4-step workup is shown. 1st step: differentiation of MUOs in clear-cut and not clear-cut carcinomas. 2nd step: definition of the histotype. 3rd step: selection of specific immunoistochemical (IHC) tissue markers based to the results of step 2. 4th step: selection of the diagnostic exploration in related organs or comprehensive imaging to confirm the diagnosis of CUP or early metastatic cancer (EM)

Taking into consideration the results described above, we designed a 4-step workup (Fig. 5).

This algorithm considers “histology” as the guiding parameter of the diagnostic cascade of MUOs, with the intent to define those with clear-cut carcinoma characteristics. This first step followed the definition of the specific tumor histotype in the two sets of MUOs. In adenocarcinomas, cytohistological features per se solved MUOs origin in 7/17 cases, identifying 41% of early metastatic cancers. If no pathological characteristic of a specific organ has been recognized, in adenocarcinomas, the expression of CK7/CK20, along with the sex and site of metastases, headed to the selection of specific IHC markers to define an alleged source tissue immunophenotype, which in turn guided targeted diagnostic investigations. This pathway led to identify 9/17 (53%) early metastatic adenocarcinomas. In squamous and neuroendocrine carcinomas, the site of metastases and the patient gender were not considered to posit a tissue of origin, and each patient was studied with PET and CT. All were diagnosed as confirmed CUP. The epithelial nature of cancers without clear features of carcinoma was tested using many different pan-cytokeratins and other markers of cell differentiation, which identified one melanoma (5.8% of early metastatic cancers).

### Comparison of clinical and pathological characteristics of confirmed CUP and early metastatic carcinomas

Of the 64 MUOs, 47 (73.4%) were confirmed CUPs, and 17 (26*.*5%) were early metastatic cancers. Histotype (*p* = 0.045), grade (*p* = 0.04), and putative immunophenotype (*p* = 0.01) were significantly different between confirmed CUP and early metastatic cancers, as shown in Table 2.

**Table 2:** Clinical and pathological comparison of confirmed carcinoma of unknown primary (CUP) and early metastatic cancers (EM)

		47 CUP		17 EM		*p* value (95% CI)
		*N*°	%	*N*°	%	
Gender	Women	26	55.31	12	70.85	*p* = 0.52
	Men	21	44.68	5	29.41	
Age	1–34	2	4.25	2	11,76	*p* = 0.39
	35–70	35	74.46	11	64.70	
	71–96	10	21.27	4	23.52	
Histotype	Adenocarcinomas	24	51.06	16	94.11	*p* = 0.041
	Squamous carcinomas	11	23.40	0	0	
	Neuroendocrine carcinomas	3	6.38	0	0	
	Sarcomatoid tumors	5	10.63	0	0	
	Undifferentiated tumors	4	8.51	1	5.88	
Grade	Low	1	2.12	5	29.41	*p* = 0.04
	Intermediate	16	34.04	3	17.64	
	High	30	63.82	9	52.94	
Putative immunophenotype of adenocarcinomas	Biliopancreatic	6	25	3	18.75	*p* = 0.01
	Breast	0	0	6	37.5	
	Gynecological	6	25	1	6.25	
	Lung	2	8.33	3	18.75	
	Thyroid	0	0	1	6.25	
	Stomach	0	0	1	6.25	
	Intestine	0	0	1	6.25	
	Undefined	10	41.66	0	0	
Site of metastases at diagnosis	Lymph node	21	44.68	3	17.64	*p* = 0.17
	Lymph node and others*	19	40.42	9	52.94	
	Others*	7	14.89	5	29.41	
Number of metastases	Single	8	17.02	4	23.52	*p* = 0.45
	Multiple	39	82.97	13	76.47	

^*^Others: visceral and/or bone and/or muscle and/or brain site of metastasis

### CUP genetic analysis by ONCOCARTA

**Fig. 6:**
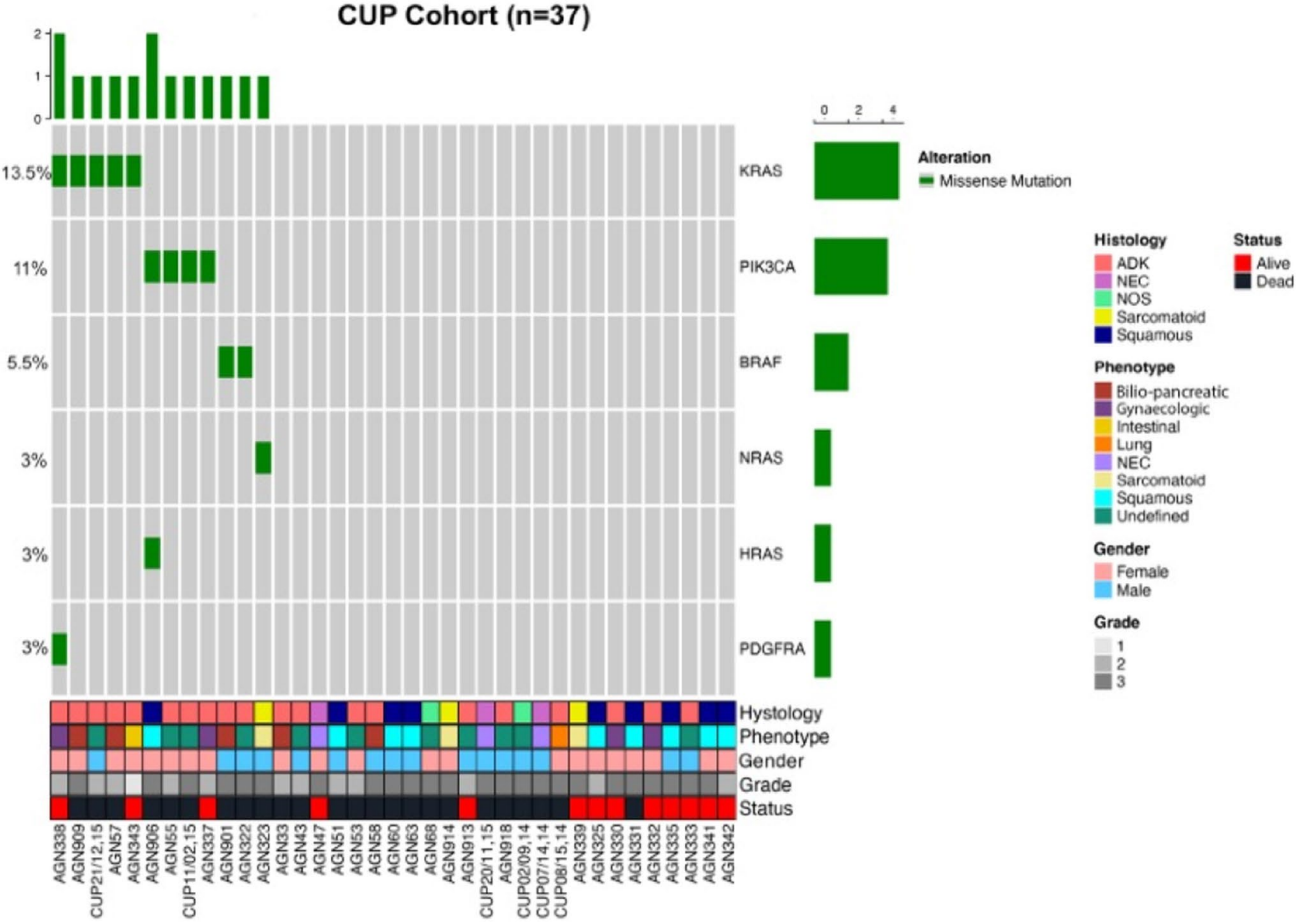
Oncoprint. The graph reports the DNA mutations identified in the cohort of 37 CUPs analyzed by targeted sequencing. Gene names and relative frequency of mutations are reported in the double y-axis. The bar graph in the top of the oncoprint defined the number of variant/sample. Histology, phenotype, gender, tumor grade, and alive status are annotated at the bottom of the plot. ADK: adenocarcinoma, NEC: neuroendocrine carcinoma, NOS: not otherwise specific or undifferentiated tumors

The oncoprint in Fig. 6 summarizes the genetic analysis by the ONCOCARTA gene panel that explores more than 230 somatic mutations across 19 actionable oncogenes frequently mutated in human cancers.

The leftover of FFPE tissue after the IHC study was adequate for molecular analyses in 37/47 CUP cases (78.7%). Twelve out of the 37 cases (36%) showed at least one mutation; 10 of these were adenocarcinomas, 1 was SSC, and 1 was sarcomatoid carcinoma. The remaining 25 CUPs did not show any detectable alterations. *KRAS* and *PI3KCA* were the most frequently mutated genes and accounted for 75% of all the identified alterations. *KRAS*, *BRAF*, *NRAS*, and *PIK3CA* mutations were found with a mutually exclusive pattern. When subdividing by CUP histological type, the adenocarcinoma was the most frequently mutated (10/12 samples); in addition, only adenocarcinomas harbored *KRAS* mutations. The most mutated immunophenotypes of adenocarcinomas were the undefined (1 *KRAS*; 2 *PIK3CA*, and 1 *BRAF*) and the biliopancreatic (2 *KRAS* and 1 *BRAF*), followed by the gynecological immunophenotype (1 case with concomitant *KRAS* and *PDGFRA* missense mutation and 1 with *PIK3CA* mutation). The pseudomyxoma peritonei with intestinal immunophenotype (AGN343) showed *KRAS* mutation. One sarcomatoid carcinoma (AGN323) showed *NRAS* mutation. One SCC showed co-occurrence of a *PIK3CA* and a *HRAS* mutation. CUPs showing NE differentiation were wild type for the evaluated genes. No specific correlation was found between specific mutations and immunophenotype, sex, histological grade, or survival status.

### Clinical impact of CUP histology and immunophenotype

It is known that CUP patients with only lymph node metastases have a better OS [11]. Thus, we focused on confirmed CUP patients and assessed the site of first metastases, the site of progression, and their possible correlation with the histotype and, in the case of adenocarcinomas, with the putative immunophenotype (Tables 3 and 4).

**Table 3:** Evolution of CUP with lymph node localization at diagnosis

Lymph node localization of CUP at diagnosis										
					No progression/lymph node progression only	Months of FU		Other organs progression/death	Months of FU	
CUP	Total *N*°		*N*°	%	*N*°	Range	Mean	*N*°	Range	Mean
Adenocarcinoma immunophenotype										
Gynecologic	6		4	66	4	11.5–78	29.6	0	–	–
Lung	2		0	0	0	–	–	0	–	–
Biliopancreatic	6		0	0	0	–	–	0	–	–
Undefined	10		4	40	1	–	104	3	13–47.2	31.4
SCC	11		7	70	3	7.4–14.5	10,1	4	18.3–49.7	26.7
NE carcinoma	3		1	33.3	1	–	65	0	–	–
Sarcomatoid carcinoma	5		3	60	1	–	9	2	5.3–11	8
Undifferentiated carcinoma	4		1	25	0	–	–	1	–	14
Total	47		20	42.5	10		43.5	10		20

*SCC* squamous cell carcinoma, *NE* neuroendocrine

**Table 4:** Evolution of CUP with any localization (with or without lymph node) at diagnosis

Any localization of CUP at diagnosis with or without lymph node metastases									
				No progression/lymph node progression only	Months of FU		Other organs progression/death	Months of FU	
CUP	Total *N*°	*N*°	%	*N*°	Range	Mean	*N*°	Range	Mean
Adenocarcinoma immunophenotype									
Gynecologic	6	2	33	0	–	–	2	1.3–8.5	4,9
Lung	2	2	100	0	–	–	2	6.0–16	11
Biliopancreatic	6	6	100	0	–	–	6	3.2–21	12,8
Undefined	10	6	60	0	–	–	6	0–18.5	6,2
SCC	11	4	30	3	3–19.5	9,3	1	–	50
NE carcinoma	3	2	66.7	0	–	–	2	3.4–18.5	10.9
Sarcomatoid carcinoma	5	2	40	1	–	3.5	1	–	4
Undifferentiated carcinoma	4	3	75	1	–	3.6	2	4.2–14.5	9
Total	47	27	57.4	5		5.4	22		13.6

*SCC* squamous cell carcinoma, *NE* neuroendocrine

**Fig. 7:**
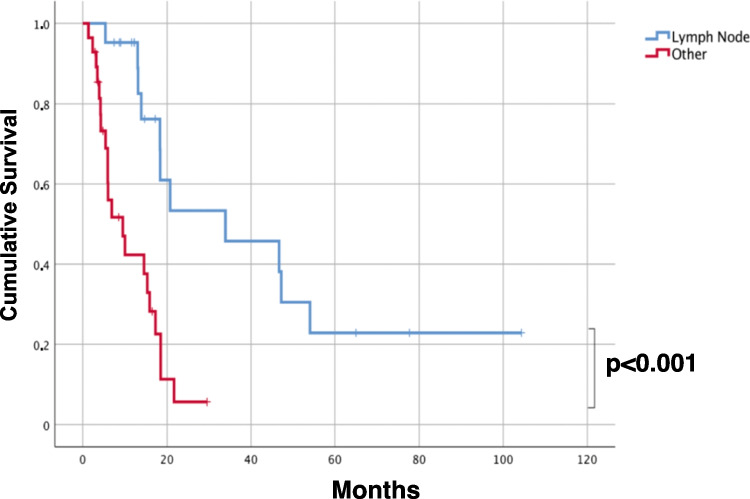
Overall survival (OS) analysis. OS survival curve for CUP patients, stratified for the site of the lesion onset (lymph nodes only versus all other sites with or without lymph node involvement). CUP patients who had only lymph node metastases at first diagnosis had a significantly longer overall survival (< 0.001) than the patients who had metastases elsewhere (visceral and/or bone and/or muscle and/or brain) with or without lymph node involvement

Specifically, squamous cell carcinoma histotype and the “gynecologic immunophenotype” within adenocarcinomas presented frequently with lymph nodes as the solely involved organs. None of the patients within the “bilio-pancreatic” and “lung immunophenotype” of adenocarcinomas presented with lymph node involvement only. Patients with “undefined immunophenotype” of adenocarcinomas, undifferentiated, and sarcomatoid carcinomas frequently progressed, independently from the anatomical site of cancer at diagnosis. Altogether, 20/47 (42.5%) confirmed CUP patients had only lymph node metastases at first diagnosis, while 27 (57.5%) had metastases elsewhere (visceral and/or bone and/or muscle and/or brain) with or without lymph node involvement (Table 3) (Supplementary Figs. 1A and B). The time to organ progression other than lymph nodes or death for the disease was 6.4 months longer in patients with lymph node metastases only, independently from the number of lymph node involved and the regional location. Finally, the patients with only lymph node metastases at first diagnosis had a significantly longer OS (< 0.001), independently again from the number of lymph node involved and the regional location (sub- or supra-diaphragmatic) (Fig. 7).

## Discussion

Using the data collected from a real-life experience, we here envisage a streamlined pathological workup to rule out CUPs in patients presenting with MUOs.

MUO reviews [12–16] and guidelines [2, 3, 6] are continuously refined, with the aim of defining a diagnostic workup capable of approximating the tissue of origin as closely as possible to better guide therapeutic decisions. With this aim, the CUPISCO trial should have produced a comprehensive genomic profile of patients carrying unfavorable CUPs, to identify possible targets of gene therapy [17]. One of the reasons for the failure of the CUPISCO study was the inadequacy of residual tissue after histopathological diagnosis for high throughput molecular analyses [18]. In the present series, the tissue leftover after immunohistochemical analyses was inadequate for molecular analyses in 21% of the CUP cases. Another reason of the CUPISCO trial failure was that 10% (13/124) of cases included among unfavorable CUP, at central review turned out as non-carcinoma malignancies [18]. In the present study, undifferentiated and sarcomatoid tumors represented 16.7% of MUOs and were mainly tested with different CK antibody panels to confirm their epithelial differentiation. The sarcomatoid tumors were all positive with at least two pan-cytokeratin antibodies and considered carcinomas, although some positivity for vimentin could be observed. On the other hand, aberrant focal expression of cytokeratin has been reported in melanoma and sarcomas [15]. The single case of melanoma, representing 1.5% of MUOs, was CK-negative and positive for Melan-A and S100. In the CUPISCO trial, the misdiagnosis of CUP due to melanomas and sarcomas represented 1.6% and 5.6%, respectively, of the failure cases [18].

The Royal Collage of Pathologists recommends then testing samples with antibodies reactive with germ cell tumors [3]. Accordingly, with NICE guidelines [2], we think that patient age, site of metastases, and serum tumor markers such as alpha-fetoprotein, human chorion gonadotropin, and lactate dehydrogenase [19, 20] may be more effective in the diagnosis of germ cell origin than tissue immunophenotyping.

Once carcinomas are identified, the final step recommended by the guidelines [3] is to determine the likely tissue of origin. Belizzi AM [21] reported that through the application of next-generation immunohistochemistry, pathologists can provide better answers than ever before. The author stressed the role of tumor morphology in leading the CUP diagnostic workflow, together with the acknowledgement that the metastatic site helps tracking the primary lesion. Above all, when lymph nodes are the exclusive site of metastases, it is essential to consider the organs that drain to those lymph nodes as a possible site of origin of the tumor [22]. Actually, the NICE guidelines [2] recommend to manage metastatic squamous carcinoma in cervical lymph nodes as being of head and neck origin and those in inguinal lymph nodes as of anal/lower gynecological tract/urological origin. Our results have shown that patient gender, together with the metastasis site, is an important leading parameter in CUP diagnosis particularly in the set of adenocarcinomas, the most frequent (62.5% of total cases, consistently with previous works [2, 3, 6]) and the most challenging histotype among MUOs. On the other hand, adenocarcinomas may show cytohistological features, for example, follicular and papillary structures or signet ring cells, which per se may be pathognomonic of tumor origin and consequently address tissue targeted markers. In our case series, these features led to identify 42.5% (7/40) of early metastatic adenocarcinomas. If no specific cytohistological patterns are present, in adenocarcinomas CK7/CK20 expression, gender and site of metastases strongly influenced the selection of tissue-specific markers, to depict a “putative immunophenotype” of tissue of origin. All women adenocarcinoma biopsies were in fact tested for ER. In CK7 + /CK20 − cases, ER positivity and GATA3 positivity in ER-negative cases primarily directed clinicians to complete radiological imaging investigations of the breast as a possible site of cancer origin, especially in patients with axillary lymph node metastases. Using these immunophenotypical markers and the cytohistological features crucial for lobular breast cancer diagnosis, 6 early metastatic breast cancers out of 64 MUO cases (9.23%) were confirmed. In the CUPISCO trial, 7.3% of the cases were not compatible with CUP because of proof or strong evidence of a breast primary [18]. In agreement with Pauli C. et al. [18], the expression of PAX8/WT1 was considered to investigate a possible gynecological origin. If primary ER + breast cancers are excluded, TTF1/napsin A were instead the key tissue markers in male CK7 + /CK20 − and in female CK + /CK20 − ER − MUO biopsies. Based on CUPISCO trial experience [18], although TTF1 expression in a metastatic setting does not unquestionably prove a primary origin in the lung, all TTF1/napsin A positive cases should be referred to radiologists to rule out lung primitivity. In the present series, the “lung immunophenotype” pinpointed 60% of early metastatic lung cancers. Finally, in 25% of the adenocarcinoma cohort, the immunophenotyping did not suggest any tissue of origin (*undefined immunophenotype*) nor any instrumental examination ratified a primary tumor, directly leading to the diagnosis of confirmed CUP.

In squamous cell carcinomas and neuroendocrine carcinomas, the use of cell differentiation markers is advised to avoid misinterpretation, especially when tumor morphology is heterogenous or poorly differentiated, because these histotypes are considered “favorable CUPs” [18].

When we analyzed whether the CUP histotype and the “putative immunophenotype” were associated with specific clinical evolution, we showed that the site of metastases at disease presentation influenced the length of time to progression. Notably, patients with adenocarcinomas of “undefined immunophenotype” and undifferentiated and sarcomatoid carcinomas frequently progressed with diffuse metastases, independently from the anatomical site of cancer at diagnosis, and could be considered in the category of “unfavorable CUPs.” On the other hand, CUP of “the gynecologic immunophenotype” and squamous carcinoma CUP presenting with only lymph node metastases at diagnosis had a longer time to progression. Wach MM et al. [23] showed that nearly two-thirds of patients undergoing axillary or inguinal lymphadenectomy for metastatic squamous carcinoma of unknown primary were alive 5 years following lymphadenectomy. Recently, Pouyiourou M et al. advocate to pursue localized treatment with surgery and/or radiotherapy in single-site and oligometastatic CUP [24]. Although we could not collect the type of treatment of all patients, we showed that CUP patients, who had only lymph node metastases at first diagnosis, had longer time to progression to other organs (6.4 months) and significantly longer overall survival (< 0.001) independently from the number of lymph nodes involved and the regional location (superficial vs deep localization and supra- vs sub-diaphragmatic).

Finally, we recently isolated stem-like cell spheres (agnospheres) from CUP specimens. The agnospheres recapitulated the IHC and molecular phenotypes of CUP and spontaneously and quickly give rise to multiple metastases after subcutaneous engraftment in mice [25]. Interestingly, the CUP samples that did not engraft stemmed from the two long survivors (AGN47 *neuroendocrine* carcinoma, OS: 65 months, and AGN913 adenocarcinoma with *undefined immunophenotype*, OS 104 months) both presented at diagnosis with metastases limited to lymph nodes [25].

## Conclusions

Standard histology is then essential to drive MUO’s diagnostic workup and, when combined with the “putative-immunophenotype” in adenocarcinomas and the metastatic pattern at disease outset, provides prognostic evidence for patients with CUP.

We acknowledge that our study has some limitations. The cohort was relatively small, and a validation cohort was not available; nevertheless, MUOs are rare entities and a careful clinic-pathological examination of these patients is of utmost importance in order not to misinterpret cases of early metastatic disease as CUPs.

These data may pave the way to further validation studies on the diagnostic workup leading to proper identification of CUPs.

## Supplementary Information

Below is the link to the electronic supplementary material.

Supplementary file1 (DOCX 534 KB)

## Data Availability

All results are reported within the text or in the supplementary material.

## References

[1] Kolling S, Ventre F, Geuna E, Milan M, Pisacane A, Boccaccio C, Sapino A, Montemurro F (2019). “Metastatic Cancer of Unknown Primary” or “Primary Metastatic Cancer”?. Front Oncol.

[2] National Institute for Health and Care Excellence (2019) Metastatic malignant disease of unknown primary origin in adults: diagnosis and management [NICE Guideline No. 104]. https://www.nice.org.uk/guidance/cg104 31846261

[3] Schofield JB, Oien K (2018) G167 Dataset for histopathological reporting of cancer of unknown primary (CUP) and malignancy of unknown primary origin (MUO)

[4] Shen Y, Chu Q, Yin X, He Y, Bai P, Wang Y, Fang W, Timko MP, Fan L, Jiang W (2021). TOD-CUP: a gene expression rank-based majority vote algorithm for tissue origin diagnosis of cancers of unknown primary. Brief Bioinform.

[5] Moran S, Martinez-Cardus A, Sayols S, Musulen E, Balana C, Estival-Gonzalez A, Moutinho C, Heyn H, Diaz-Lagares A, de Moura MC, Stella GM, Comoglio PM, Ruiz-Miro M, Matias-Guiu X, Pazo-Cid R, Anton A, Lopez-Lopez R, Soler G, Longo F, Guerra I, Fernandez S, Assenov Y, Plass C, Morales R, Carles J, Bowtell D, Mileshkin L, Sia D, Tothill R, Tabernero J, Llovet JM, Esteller M (2016). Epigenetic profiling to classify cancer of unknown primary: a multicentre, retrospective analysis. Lancet Oncol.

[6] Fizazi K, Greco FA, Pavlidis N, Daugaard G, Oien K, Pentheroudakis G, Committee EG (2015). Cancers of unknown primary site: ESMO Clinical Practice Guidelines for diagnosis, treatment and follow-up. Ann Oncol.

[7] Pauli C, Bochtler T, Mileshkin L, Baciarello G, Losa F, Ross JS, Pentheroudakis G, Zarkavelis G, Yalcin S, Ozguroglu M, Beringer A, Scarato J, Mueller-Ohldach M, Thomas M, Moch H, Kramer A (2021). A challenging task: identifying patients with cancer of unknown primary (CUP) according to ESMO guidelines: the CUPISCO trial experience. Oncologist.

[8] Benvenuti S, Milan M, Geuna E, Pisacane A, Senetta R, Gambardella G, Stella GM, Montemurro F, Sapino A, Boccaccio C, Comoglio PM (2020). Cancer of unknown primary (CUP): genetic evidence for a novel nosological entity? A case report. EMBO Mol Med.

[9] Elston CW, Ellis IO (1991). Pathological prognostic factors in breast cancer. I The value of histological grade in breast cancer: experience from a large study with long-term follow-up. Histopathology.

[10] Cancer NCCf (2010) Diagnosis and management of metastatic malignant disease of unknown primary origin. pp. 22259823

[11] Hemminki K, Bevier M, Hemminki A, Sundquist J (2012). Survival in cancer of unknown primary site: population-based analysis by site and histology. Ann Oncol.

[12] Bochtler T, Löffler H, Krämer A (2018) Diagnosis and management of metastatic neoplasms with unknown primarySeminars in diagnostic pathology. Elsevier, 199–206 10.1053/j.semdp.2017.11.013 29203116

[13] Kato S, Alsafar A, Walavalkar V, Hainsworth J, Kurzrock R (2021) Cancer of unknown primary in the molecular era Trends in cancer 10.1016/j.trecan.2020.11.002 PMC8062281 33516660

[14] Laprovitera N, Riefolo M, Ambrosini E, Klec C, Pichler M, Ferracin M (2021). Cancer of unknown primary: challenges and progress in clinical management. Cancers.

[15] Lin F, Liu H (2014). Immunohistochemistry in undifferentiated neoplasm/tumor of uncertain origin. Arch pathol lab med.

[16] Qaseem A, Usman N, Jayaraj JS, Janapala RN, Kashif T (2019) Cancer of unknown primary: a review on clinical guidelines in the development and targeted management of patients with the unknown primary site Cureus 11 10.7759/cureus.5552 PMC6820325 31695975

[17] Ross JS, Sokol ES, Moch H, Mileshkin L, Baciarello G, Losa F, Beringer A, Thomas M, Elvin JA, Ngo N (2021). Comprehensive genomic profiling of carcinoma of unknown primary origin: retrospective molecular classification considering the CUPISCO study design. Oncologist.

[18] Pauli C, Bochtler T, Mileshkin L, Baciarello G, Losa F, Ross JS, Pentheroudakis G, Zarkavelis G, Yalcin S, Özgüroğlu M (2021). A challenging task: identifying patients with cancer of unknown primary (CUP) according to ESMO guidelines: the CUPISCO trial experience. oncologist.

[19] Gilligan T, Seidenfeld J, Basch E, Einhorn L, Fancher T, Smith D, Stephenson A, Vaughn D, Cosby R, Hayes D (2010). American Society of Clinical O: American Society of Clinical Oncology clinical practice guideline on uses of serum tumor markers in adult males with germ cell tumors. J Clin Oncol.

[20] Schmoll H, Souchon R, Krege S, Albers P, Beyer J, Kollmannsberger C (2004). European Germ Cell Cancer Consensus Group. European consensus on diagnosis and treatment of germ cell cancer: a report of the European Germ Cell Cancer Consensus Group (EGCCCG). Ann Oncol.

[21] Bellizzi AM (2020). An algorithmic immunohistochemical approach to define tumor type and assign site of origin. Adv Anat Pathol.

[22] Shao Y, Liu X, Hu S, Zhang Y, Li W, Zhou X, Wang Q, Hou Y, Chen Y, Wang Y, Wang Y, Luo Z, Hu X (2020). Sentinel node theory helps tracking of primary lesions of cancers of unknown primary. BMC Cancer.

[23] Wach MM, van Beek E, Ayabe R, Ruff S, Brown Z, Goldman DA, Zambirinis CP, Gholami S, Pulitzer M, Hernandez J (2018). Metastatic squamous cell carcinoma of known and unknown primary origin treated with axillary or inguinal lymphadenectomy. The American Journal of Surgery.

[24] Pouyiourou M, Wohlfromm T, Kraft B, Hielscher T, Stichel D, von Deimling A, Delorme S, Endris V, Neumann O, Stenzinger A (2021). Local ablative treatment with surgery and/or radiotherapy in single-site and oligometastatic carcinoma of unknown primary. Eur J Cancer.

[25] Verginelli F, Pisacane A, Gambardella G, D'Ambrosio A, Candiello E, Ferrio M, Panero M, Casorzo L, Benvenuti S, Cascardi E, Senetta R, Geuna E, Ballabio A, Montemurro F, Sapino A, Comoglio PM, Boccaccio C (2021). Cancer of unknown primary stem-like cells model multi-organ metastasis and unveil liability to MEK inhibition. Nat Commun.

