# Correction to: Real-world histopathological approach to malignancy of undefined primary origin (MUO) to diagnose cancers of unknown primary (CUPs)

**DOI:** 10.1007/s00428-022-03475-5

**Published:** 2022-12-17

**Authors:** Alberto Pisacane, Eliano Cascardi, Enrico Berrino, Alessio Polidori, Ivana Sarotto, Laura Casorzo, Mara Panero, Carla Boccaccio, Federica Verginelli, Silvia Benvenuti, Miriam Dellino, Paolo Comoglio, Filippo Montemurro, Elena Geuna, Caterina Marchiò, Anna Sapino

**Affiliations:** 1 Candiolo Cancer Institute, FPO-IRCCS, 10060 Candiolo, Turin, Italy; 2 Department of Medical Sciences, University of Turin, 10100 Turin, Italy; 3 Department of Oncology, University of Turin Medical School, 10060 Candiolo, Turin, Italy; 4 Department of Biomedical Sciences and Human Oncology, University of Bari “Aldo Moro”, Bari, Italy; 5 IFOM, FIRC Institute of Molecular Oncology, 20019 Milan, Italy

**Correction to: Virchows Archiv**

10.1007/s00428-022-03435-z

In the original published version of the above article, the affiliation, Candiolo Cancer Institute, FPO-IRCCS, 10060 Candiolo Turin, Italy, was not presented in the authors’ information of the co-author Carla Boccaccio. This is now presented correctly.

The original article has been corrected.

