# Supplementary material for: Real-world histopathological approach to malignancy of undefined primary origin (MUO) to diagnose cancers of unknown primary (CUPs)
Source: Virchows Arch. 2022 Nov 8;482(3):463–75. doi: 10.1007/s00428-022-03435-z (PMC9640798; doi:10.1007/s00428-022-03435-z)
Supplement: Supplementary file 1 — Supplementary file1 (DOCX 534 KB) [file 428_2022_3435_MOESM1_ESM.docx]

**Supplementary Figure 1A**


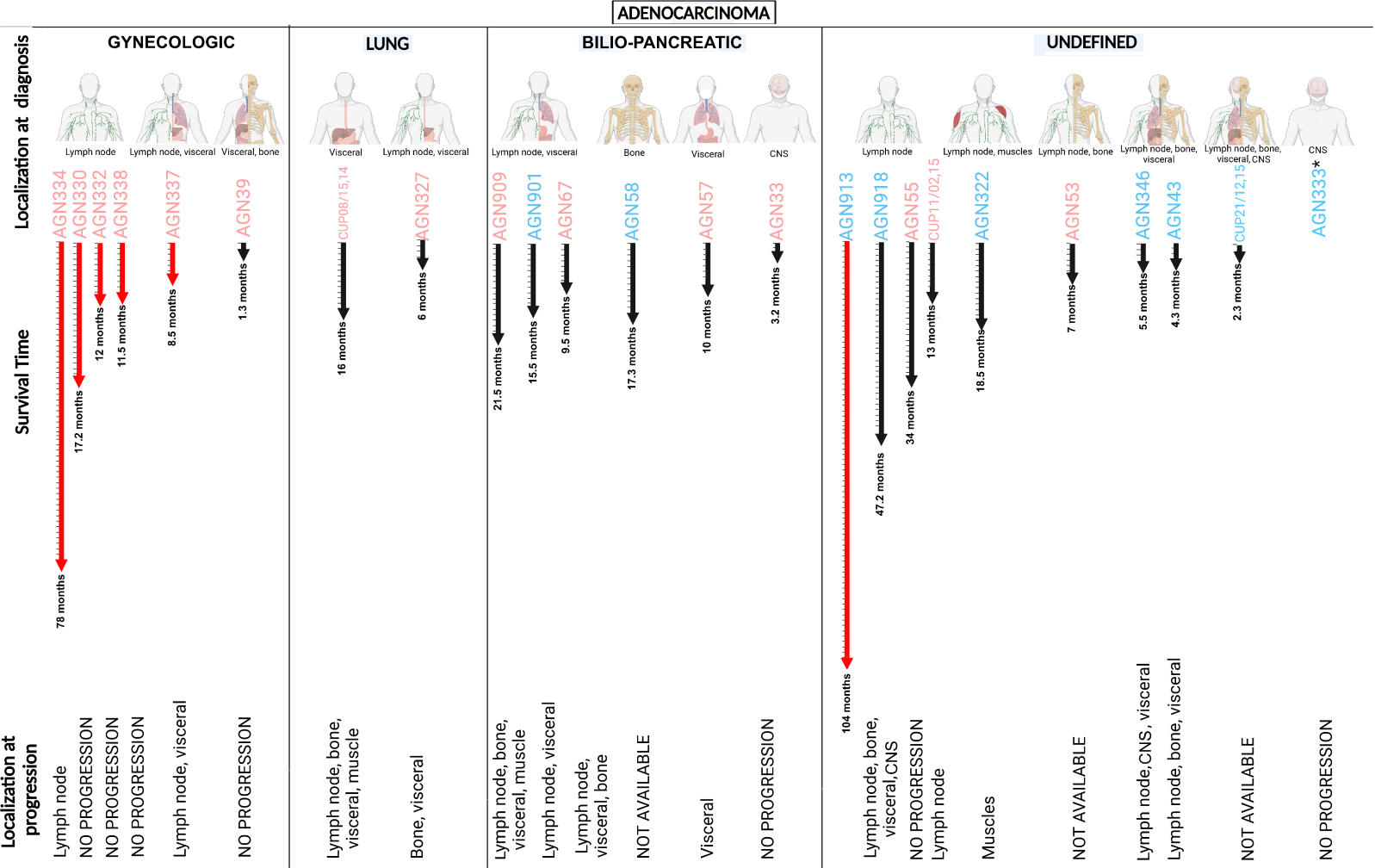


**Supplementary Figure 1B**


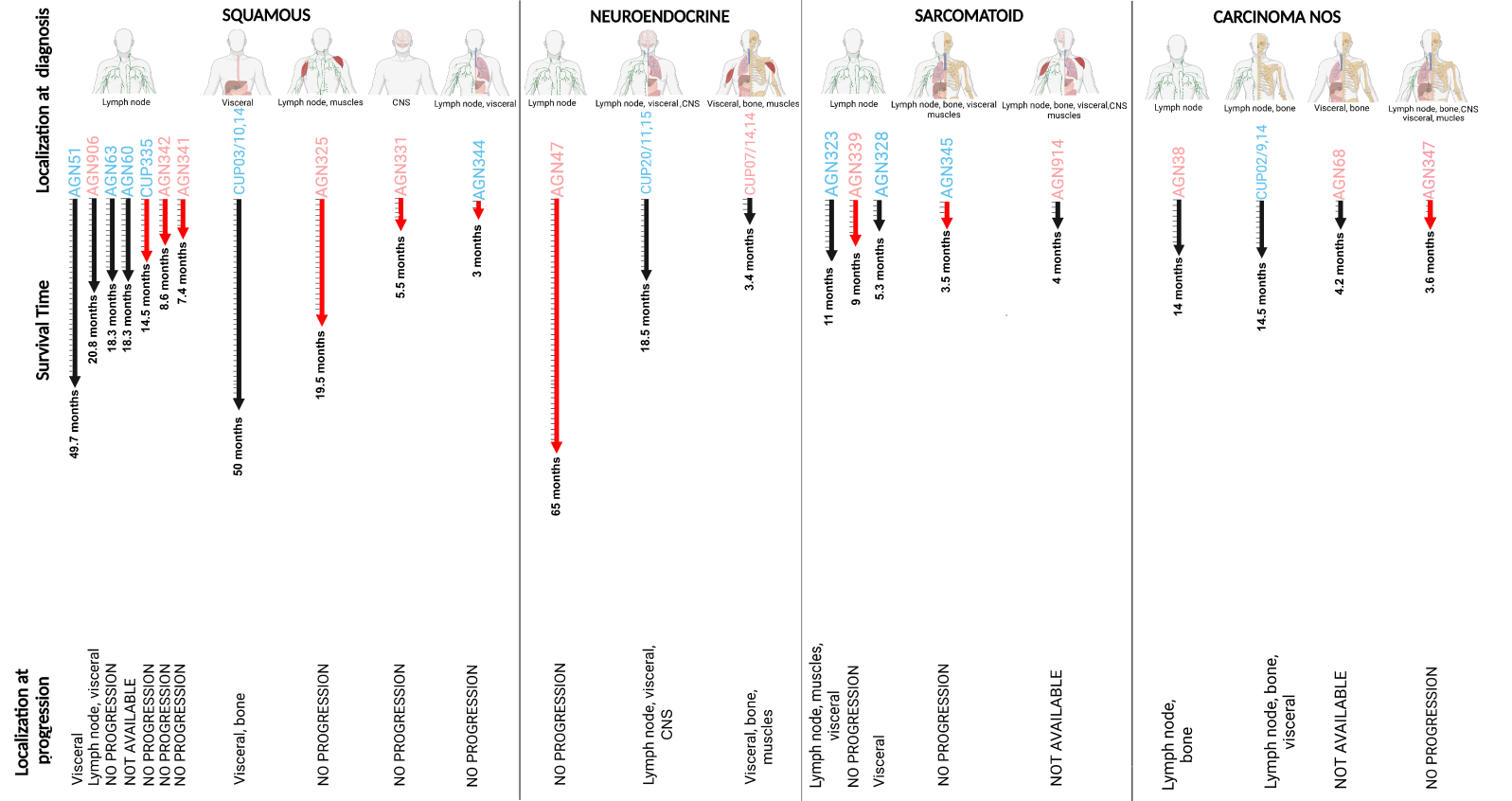
**Supplementary Figure 1. Graphical representation of disease progression of patients with (A) adenocarcinomas and (B) CUPs not-adenocarcinoma.** *Schematic Localization of the tumor* *at diagnosis for each patient. The timeline scale represents the shortest time interval (40 days) of follow up. The progression and the site of progression is reported. Red timelines: time of follow-up (months) of alive patients; black timelines: time of follow-up (months) of dead patients. * the patient died after 2 weeks.*
